# Understanding and Measuring Resilience: From Bench to Bedside

**DOI:** 10.1093/geroni/igaf122.1056

**Published:** 2025-12-31

**Authors:** Chenkai Wu, Heather Whitson

## Abstract

This symposium examines the multifaceted nature of resilience across diverse clinical contexts, bridging the gap between theoretical frameworks and practical applications. Resilience, the capacity to withstand and recover from stressors, is a critical concept in aging research and clinical practice, impacting patient outcomes across various health conditions. The first presentation explores the role of physical resilience in patient satisfaction following total knee replacement, demonstrating how monitoring post-surgical physical function trajectories can optimize rehabilitation and enhance long-term patient-centered outcomes. Moving from planned surgical interventions to traumatic injuries, the second and third presentations delve into the dynamics of resilience in hip fracture recovery. One presentation deconstructs physical resilience into its fundamental components—resistance and recovery—quantifying their impact on mortality. The other examines frailty trajectories as markers of resilience, highlighting their predictive value for post-fracture outcomes. Collectively, these three presentations illustrate how resilience can be operationalized and measured using longitudinal data and advanced statistical methods. By integrating insights from electronic health records, patient-reported outcomes, and functional assessments, this symposium aims to provide a comprehensive understanding of resilience, from its conceptualization to its clinical application. Attendees will gain insights into identifying individuals with lower resilience and developing targeted interventions to improve recovery and survival. This symposium bridges the gap between research and clinical practice, fostering a deeper understanding of resilience and its potential to enhance patient care.

